# An SEM-ANN approach to evaluate public awareness about COVID, A pathway toward adaptation effective strategies for sustainable development

**DOI:** 10.3389/fpubh.2022.1046780

**Published:** 2022-10-19

**Authors:** Muhammad Tayyab Sohail, Minghui Yang, Petra Maresova, Sohaib Mustafa

**Affiliations:** ^1^School of Public Administration, Xiangtan University, Xiangtan, China; ^2^South Asia Research Center, School of Public Administration, Xiangtan University, Xiangtan, China; ^3^International Business School, Guangzhou City University of Technology, Guangzhou, China; ^4^Research Center for Accounting and Economic Development of Guangdong-Hong Kong-Macao Greater Bay Area, Guangdong University of Foreign Studies, Guangzhou, China; ^5^Faculty of Informatics and Management, University of Hradec Kralove, Hradec Kralove, Czechia; ^6^Malaysia-Japan International Institute of Technology, Universiti Teknologi Malaysia, Kuala Lumpur, Malaysia; ^7^College of Economics and Management, Beijing University of Technology, Beijing, China

**Keywords:** public, COVID-19, Pakistan, health, SEM-ANN, social distance, protective measures, public awareness about COVID-19

## Abstract

This study was conducted to evaluate public awareness about COVID with aimed to check public strategies against COVID-19. A semi structured questionnaire was collected and the data was analyzed using some statistical tools (PLS-SEM) and artificial neural networks (ANN). We started by looking at the known causal linkages between the different variables to see if they matched up with the hypotheses that had been proposed. Next, for this reason, we ran a 5,000-sample bootstrapping test to assess how strongly our findings corroborated the null hypothesis. PLS-SEM direct path analysis revealed HRP -> PA-COVID, HI -> PA-COVID, MU -> PA-COVID, PM -> PA-COVID, SD -> PA-COVID. These findings provide credence to the acceptance of hypotheses H1, H3, and H5, but reject hypothesis H2. We have also examined control factors such as respondents' age, gender, and level of education. Age was found to have a positive correlation with PA-COVID, while mean gender and education level were found to not correlate at all with PA-COVID. However, age can be a useful control variable, as a more seasoned individual is likely to have a better understanding of COVID and its effects on independent variables. Study results revealed a small moderation effect in the relationships between understudy independent and dependent variables. Education significantly moderates the relationship of PA-COVID associated with MU, PH, SD, RP, PM, PA-COVID, depicts the moderation role of education on the relationship between MU^*^Education->PA-COVID, HI^*^Education->PA.COVID, SD^*^Education->PA.COVID, HRP^*^Education->PA.COVID, PM^*^Education -> PA.COVID. The artificial neural network (ANN) model we've developed for spreading information about COVID-19 (PA-COVID) follows in the footsteps of previous studies. The root means the square of the errors (RMSE). Validity measures how well a model can predict a certain result. With RMSE values of 0.424 for training and 0.394 for testing, we observed that our ANN model for public awareness of COVID-19 (PA-COVID) had a strong predictive ability. Based on the sensitivity analysis results, we determined that PA. COVID had the highest relative normalized relevance for our sample (100%). These factors were then followed by MU (54.6%), HI (11.1%), SD (100.0%), HRP (28.5%), and PM (64.6%) were likewise shown to be the least important factors for consumers in developing countries struggling with diseases caused by contaminated water. In addition, a specific approach was used to construct a goodness-of-fit coefficient to evaluate the performance of the ANN models. The study will aid in the implementation of effective monitoring and public policies to promote the health of local people.

## Introduction

It believes that the public's concern for the environment may be growing in the wake of the COVID-19 pandemic (1). Long-term rehabilitation plans following the COVID-19 catastrophe require insights into public awareness levels, which we evaluate by their behavior (2). For instance, when public opinion moves toward a more protective stance toward COVID, it has a notable and beneficial influence on their lifestyle choices. Large-scale changes in human behavior are a direct result of the COVID-19 disaster. Because we are all strongly encouraged, if not compelled, to stay and work at home, not only have international transport flows decreased but so have traffic flows within the country (3). Attitudes regarding COVID shift as our habits evolve. The presence of an urban environment is essential to local resilience throughout this crisis since it promotes both physiological and psychological health (4–15). The quantity of media coverage matters, but not as much as other factors such as public opinion, emotions, the pollution's physical qualities, the economy, and location (7, 16–22). Based on the message's substance and phrasing, the credibility of the information provider, and the characteristics of the audience, media coverage can either increase or decrease public support for aggressive action against COVID (23). The emerging infectious illnesses represent a significant threat to human life and are extremely challenging to contain, making the rising frequency of public health catastrophes involving them a noteworthy issue (24). In turn, new demands for emergency management have emerged due to the unpredictability and complexity of these public health emergencies. Governments are faced with significant problems in the face of this new infection, including the need to implement new laws, provide aid to vulnerable groups and individuals, make progress toward attaining sustainable development objectives, and discover innovative approaches to obtain outcomes (25–27). As a result, this served as a sobering reminder that resolving a public health disaster of this magnitude is a difficult and time-consuming endeavor. During a public health emergency, it is essential to grasp how the public receives, processes, and reacts to news of the outbreak (28). Awareness of the hazards and uncertainties of developing infectious illnesses may lead to either constructive (e.g., practicing personal hand hygiene and avoiding large meetings) or disruptive (e.g., panic purchasing and embracing experimental therapies) behavior (29, 30). Unsubstantiated claims about cures for COVID-19 have been widely disseminated on social media, adding to public anxiety and concern (31, 32). As an added complication, public reactions to an outbreak may vary from one country to the next. Those in Asia started wearing masks at the first sign of the COVID-19 pandemic, while people in Europe and North America were against it (33). There is consistency between official health agency messages and available research about the efficacy of masks in protecting against COVID-19 (34). Better educate the government on risk communication and relevant official guidelines (35), it is important to comprehend why and how the public reacts to information linked to COVID-19. A growing body of research (36, 37) highlights the importance of data gleaned from online searches in the context of public health crises. Internet monitoring, in contrast to conventional surveys, may monitor public reactions to outbreaks in real-time and is less susceptible to recollection bias (38). Despite these benefits, internet surveillance to monitor public reactions and rumors during an epidemic has received surprisingly little study. The objectives of this study were (i) A SEM-ANN approach to determine public awareness about COVID-19, A pathway toward a healthy life. (ii) Public strategies against COVID-19. (iii) How public awareness about COVID-19 may help for Mask Usage (MU), Personal Hygienic (PH), Social Distance (SD), Health Risk Perception (HRP), Protective Measures (PM). (iv) Education may have moderate effects between DV and IV. On the base of our literature review, w develops the following hypothesis.

- **H1**, Mask Usage among people is directly associated with public awareness about COVID.
- **H1a**, Education plays a moderation role between Mask Usage and awareness about COVID.
- **H2**, Personal Hygienic among people is directly associated with public awareness about COVID.
- **H2a**, Education plays a moderation role between Personal Hygienic and awareness about COVID.
- **H3**, Social Distance among people is directly associated with public awareness about COVID.
- **H3a**, Education plays a moderation role between Social Distance and awareness about COVID.
- **H4**, Health Risk Perception among people is directly associated with public awareness about COVID.
- **H4a**, Education plays a moderation role between Health Risk Perception and awareness about COVID.
- **H5**, Protective Measures among people are directly associated with public awareness about COVID.
- **H5a**, Education plays a moderation role between Protective Measures and awareness about COVID.

**Figure 1:**
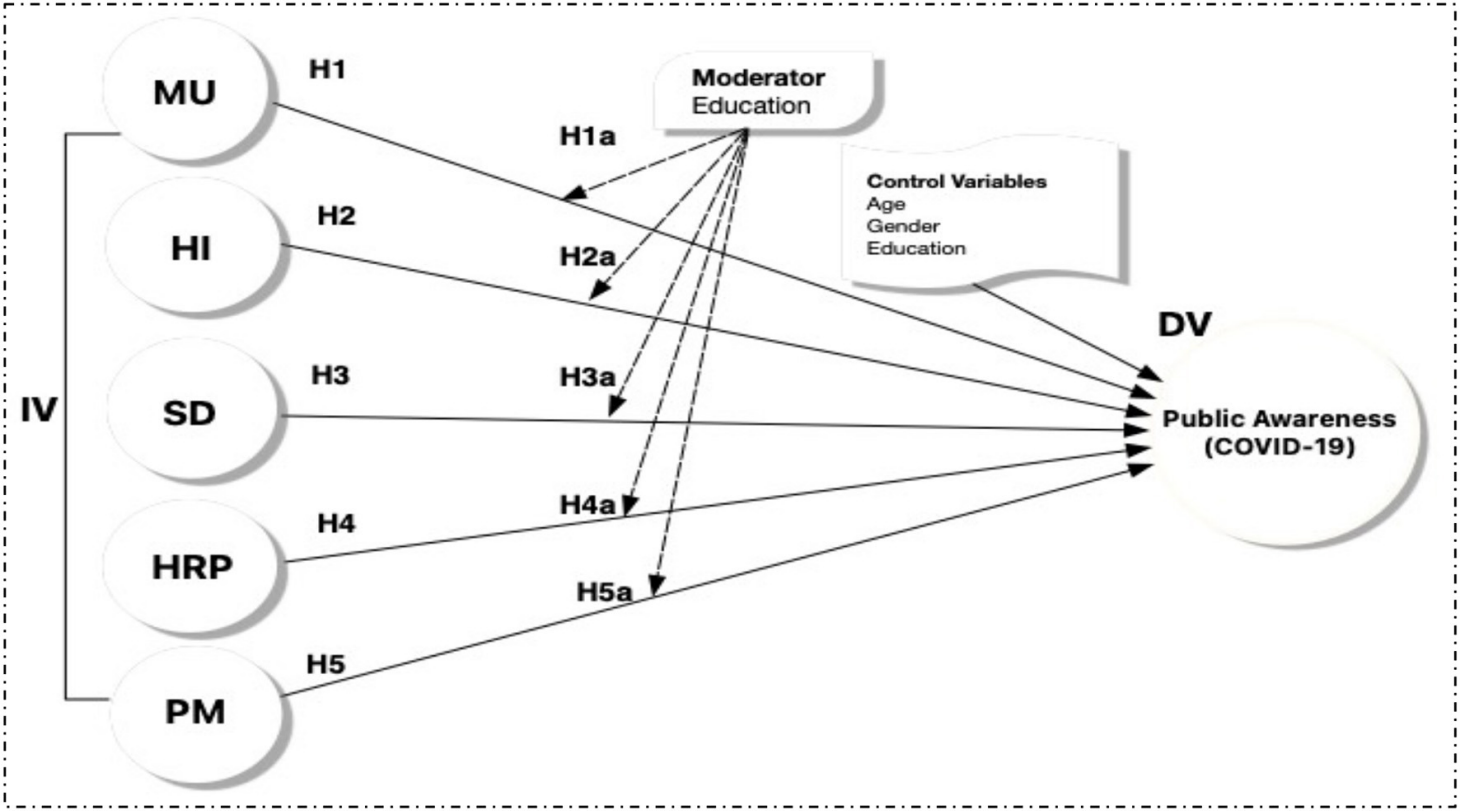
Hypothesis model: MU, mask usage; PH, personal hygienic; SD, social distance; HRP, health risk perception; PM, protective measures; PA-COVID, public awareness about COVID-19.

Moderators are essential demographic features of a sample that may strengthen the relationship between the independent and dependent variables, and they include age, gender, and level of education (39–41). According to studies conducted in Pakistan, environmental literacy among women has little effect on their propensity to purchase green products, whereas among men it has a significant bearing. As a result, we suggest that there is a significant moderation effect of education on the relationship between Mask Usage (MU), Personal Hygienic (PH), Social Distance (SD), Health Risk Perception (HRP), Protective Measures (PM), Public Awareness about COVID-19 (PA-COVID) (Figure 1).

## Materials and methods

### Study area

**Figure 2:**
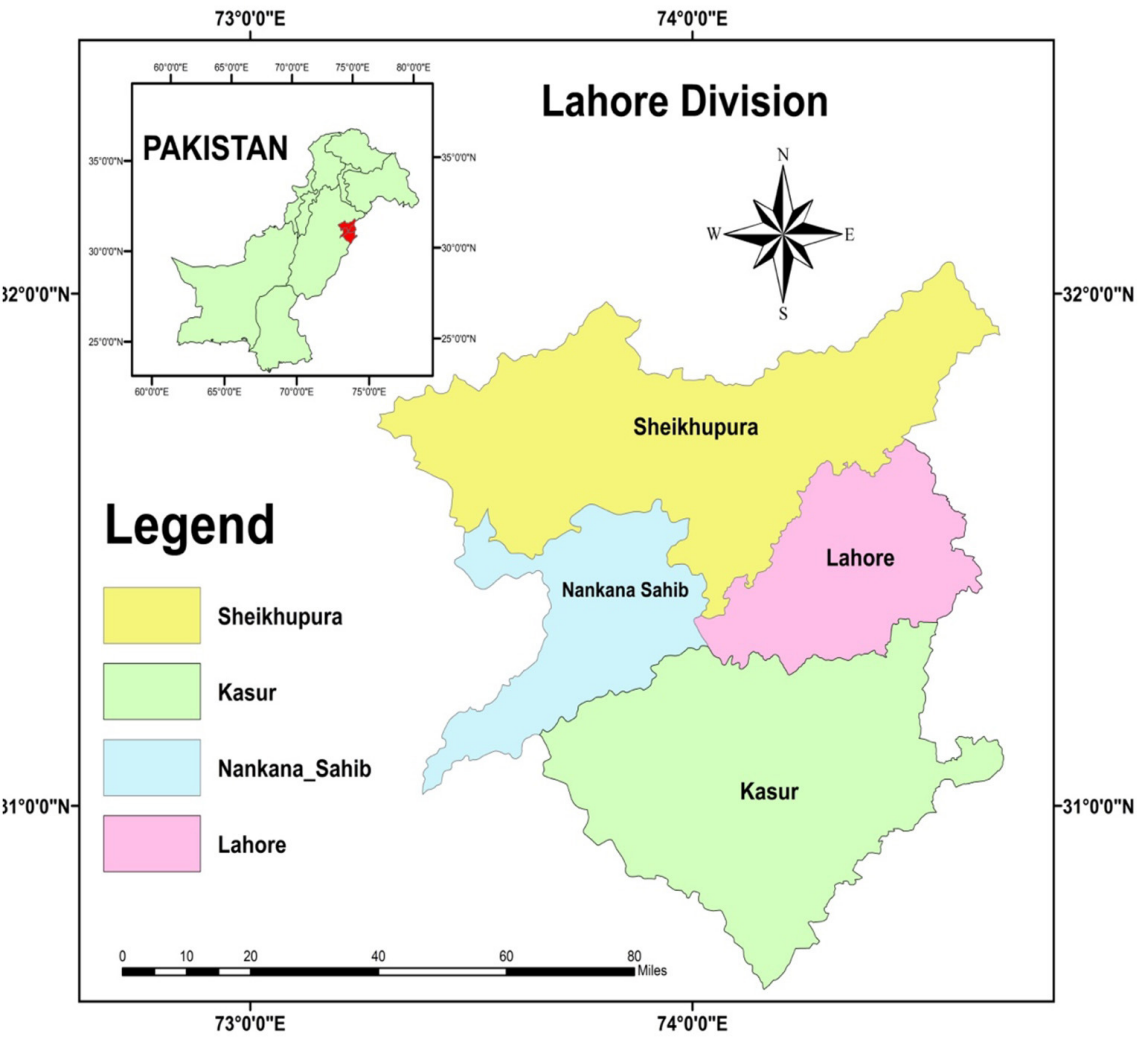
Study area.

As a sub-district of Pakistan's Punjab Province, Lahore is home to a sizable population center. Those districts are Kasur, Lahore, Nankana Sahib, and Sheikhupura (Figure 2). Lahore is second largest city of Pakistan, capital city of Punjab province and education hub of Pakistan. Lahore city and surroundings always very importance for Pakistan, this area can be game changer for rest of country to control COVID. It is the responsibility of the Commissioner to oversee operations in the Lahore Division. There are a total of four Additional Commissioners that work under the Commissioner. There is a Deputy Commissioner in charge of each division. This level of government was eliminated as part of the changes in the year 2000 but was reinstated in the year 2008. The Lahore Division was a sub-division of the Punjab Province in British India.

### Data sources and data preparation

Researchers used interviews and focus groups to learn about people's personal experiences with COVID and strategies for coping with the virus. The researchers used a semi-structured questionnaire based on prior research but tailored to the target population. People were told that their responses would be used exclusively for research reasons and that they were under no obligation to provide them following basic research ethics (42). In all, 920 completed questionnaires were collected from the research area, and a pilot study was conducted to verify the reliability of the data and eliminate sampling error. PLS-SEM and SPSS 24 were then used to analyze the data once it had been collected. Data was analyzed using PLS-SEM since this method of evaluating correlations between components is among the most effective for predicting outcomes. There has been a widespread recommendation that this technique is used for predicting and assessing explanatory variables to account for the greatest possible variance.

### Partial least squares, structural equation models (PLS-SEM)

PLS-SEM is widely recommended for predicting and assessing explanatory factors to account for the largest potential variance in results since it is one of the most effective approaches for doing so (43). PLS-SEM allows a smaller sample size to be used while still producing high-quality results, making it a preferable method. Further, it can process all models simultaneously, both internally and externally. The collected data may also be used to analyze sophisticated route models (44). Researchers have shown that the PLS-SEM method has certain promising applications in management science (39). This suggests that the PLS-SEM methodology is the most appropriate for this investigation. Because the model has non-linear account interactions, a two-stage analysis is more fruitful. To guarantee the precision and consistency of the construct evaluations, a PLS-based route modeling strategy is tested in two separate ways. First, the validity and reliability of convergent validity are assessed, and then the structural model used to create an internal model or link between the latent components is assessed.

### Artificial neural network (ANN)

Software that can simulate non-linear statistical data is called an “artificial neural network,” or “ANN.” Several training sessions might improve its performance (39–41). ANN may be used to not only predict but also organize large amounts of data. When compared to other multivariate models, the ANN model excels in making predictions. But “BLACKBOX” is not a great tactic to utilize when doing a hypothesis test. Because of this, professionals think it may be used in tandem with SEM to get a more precise outcome. For this reason, ANN is especially helpful when working with little data or flawed assumptions, as it does not rely on multivariate assumptions (3–14, 16–41).

### Geographical information system (ArcGIS)

In the cloud, ArcGIS Online provides mapping and analysis services. You may use it for collaborative mapmaking, data analysis, and sharing. Download apps tailored to your workflow, global maps and data, and field-ready mobile apps. The local and global locations of the research area are crucial pieces of information to have. ArcGIS was used to develop a study area map so that readers could easily understand the study area. Under this research, the Lahore division was selected to check public awareness about COVID-19 and how they perceive it, Lahore division consists of four districts of Punjab province named Lahore, Kasur, Sheikhupura, and Nankana Sahib (Figure 2).

## Results and discussion

**Figure 3:**
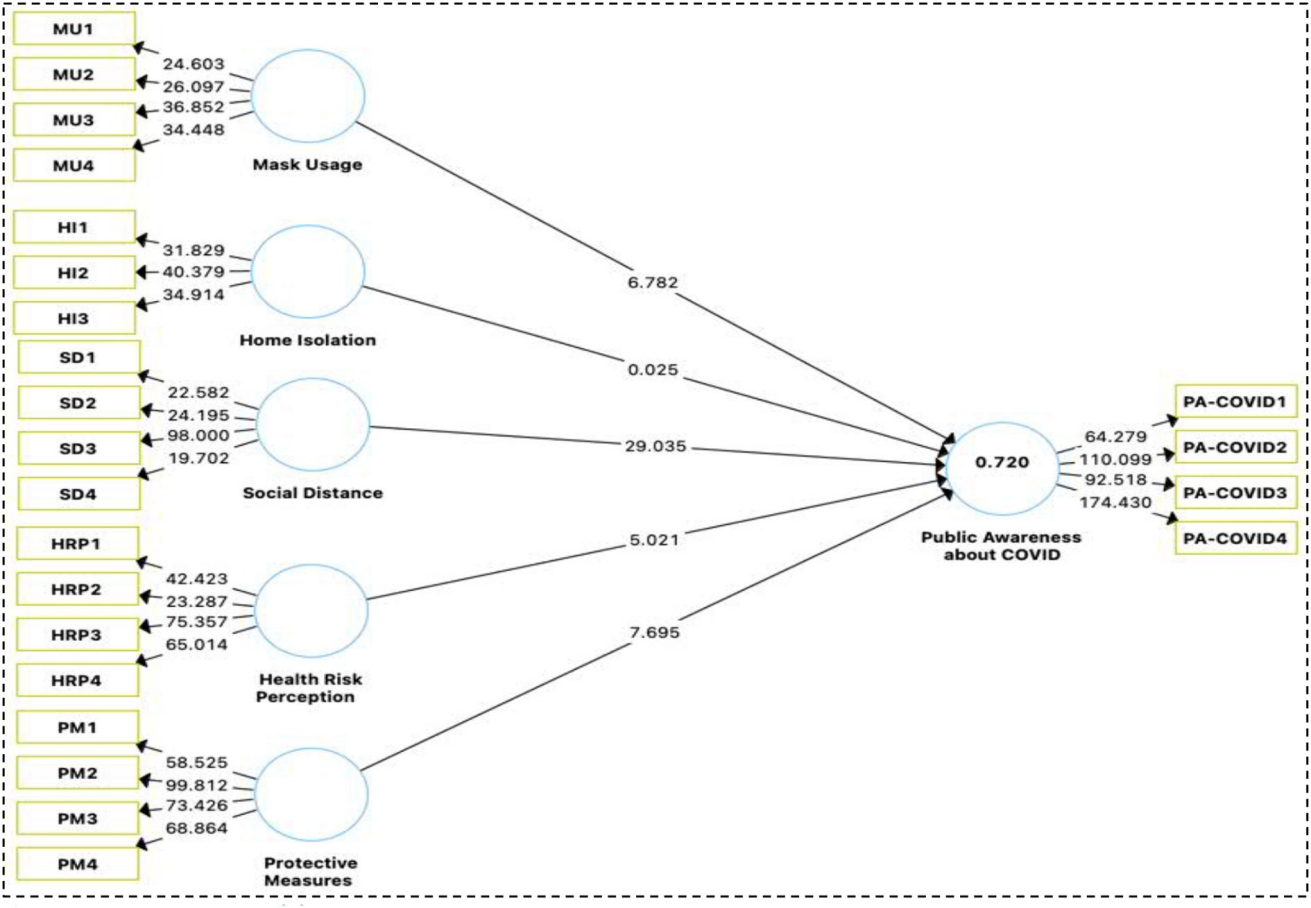
Measurement model.

This study was carried out in the Lahore division of Punjab province Pakistan. This research is based on people's own experience with COVID and how it has impacted their decision-making Mask Usage (MU), Personal Hygienic (PH), Social Distance (SD), Risk Perception (RP), Protective Measures (PM), Public Awareness about COVID-19 (PA-COVID). These results were consistent with the attribute of the sample response of gender, age, and education. In all, 920 responses were gathered for further examination of the interplay between the DV, IV, Moderator, and control variables. Researchers think that certain multivariate assumptions should be verified before embarking on a multivariate inquiry (39). Consider both the convergent and discriminant validity of the indicators and constructs in your examination of measurement models (43). To ensure that the construct indicators give a valid assessment of the research variables, we put them through a battery of tests. It was possible to ascertain the instrument's reliability using Cronbach's alpha and item loading. Measures of the extent to which the latent concept accounts for variation in indicators include the average variance extracted (AVE) and the composite reliability (CR). The average variance in expenditures is often shortened to AVE, whereas the coefficient of variation is often shortened to CR. To determine an item's trustworthiness, we look at its factor loadings on the related structures (Table 1 and Figure 3). For an outside loading to be regarded as significant it must be more than or equal to 0.6 for a given component (43). For an increased sense of trustworthiness, it is suggested that the value of Cronbach's Alpha for all structures be above or very close to the recommended cutoff of 0.7. Because of this, the results are more likely to be accurate (45). Cronbach's alpha was not the sole measure used, the composite dependability (CR) of the various structures was also calculated. The conventional approach was abandoned in favor of this one (45). In addition, these conclusions are bolstered by the strong reliability scores of the studies, all of which are more than 0.7.The Cronbach's Alpha values this research variables are mask usage (MU) *α* = 0.737, home isolation (HI) *α* = 0.698, social distance (SD) *α* = 0.791, health risk perception (HRP) *α* = 0.858, protective measures (PM) *α* = 0.885, Public Awareness about COVID-19 *α* = 0.920. As can be seen in Table 1 the AVE convergent validity estimations were either more than 0.50 or equal to it (43, 44). These results show that the dataset has adequate information for further research.

**Table 1:** Reliability and validity analysis.

**Variables**	**Items**	**Loadings**	***T*** **-value**	**VIF**	**α**	**CR**	**AVE**
Mask usage	MU1	0.719	24.603	1.486	0.737	0.832	0.553
	MU2	0.720	26.097	1.513			
	MU3	0.780	36.852	1.528			
	MU4	0.754	34.448	1.255			
Home isolation	HI1	0.763	31.829	1.257	0.698	0.833	0.624
	HI2	0.815	40.379	1.489			
	HI3	0.791	34.914	1.432			
Social distance	SD1	0.728	22.582	1.902	0.791	0.842	0.573
	SD2	0.752	24.195	2.114			
	SD3	0.831	98.000	1.196			
	SD4	0.710	19.702	1.872			
Health risk perception	HRP1	0.833	42.423	1.908	0.858	0.905	0.705
	HRP2	0.733	23.287	1.495			
	HRP3	0.898	75.357	3.079			
	HRP4	0.883	65.014	3.147			
Protective measures	PM1	0.837	58.525	2.128	0.885	0.920	0.743
	PM2	0.890	99.812	2.504			
	PM3	0.856	73.426	2.301			
	PM4	0.863	68.864	2.395			
Public awareness about COVID-19	PA-COVID1	0.882	64.279	3.527	0.920	0.943	0.806
	PA-COVID2	0.901	110.099	3.383			
	PA-COVID3	0.875	92.518	2.970			
	PA-COVID4	0.933	174.430	4.837			

The suggested model's discriminant validity is evaluated using the Fornell-Larcker criterion and heterotrait-monotrait (HTMT) ratios (43, 44). In Table 2, the most significant correlations between each set of variables suggest that Fornell-Larcker requirements have been met to prove discriminant validity (46). The HTMT ratio methodology, developed by Henseler et al. (47), is a novel method for establishing the existence or absence of discriminant validity. The researchers argued that, even if the Fornell-Larcker criteria were suitable for measuring discriminant validity, they would not be able to differentiate between the lack and presence of discriminant validity. This led to the incorporation of the HTMT into the procedure of evaluating the discriminant validity. The HTMT values for all of the criteria that were studied are listed in Table 3 below. The discriminant validity of the variables is confirmed by the fact that all of their HTMT values are less than 0.90, which means the experiment was a success and met all of the conditions (47).

**Table 2:** Fornell-Larcker criterion.

**Variables**	**Mean**	**STDEV**	**HRP**	**HI**	**MU**	**PM**	**PA-COVID**	**SD**
HRP	0.904	0.006	0.839					
HI	0.833	0.010	0.316	0.790				
MU	0.832	0.012	0.191	0.315	0.744			
PM	0.920	0.005	0.657	0.369	0.319	0.862		
PA-COVID	0.943	0.004	0.234	0.394	0.544	0.511	0.898	
SD	0.842	0.016	0.272	0.444	0.479	0.450	0.809	0.757

**Table 3:** HTMT ratio.

**Variables**	**HRP**	**HI**	**MU**	**PM**	**PA-COVID**	**SD**
HRP						
HI	0.412					
MU	0.230	0.432				
PM	0.758	0.470	0.381			
PA-COVID	0.258	0.492	0.637	0.563		
SD	0.328	0.605	0.536	0.480	0.766	

**Figure 4:**
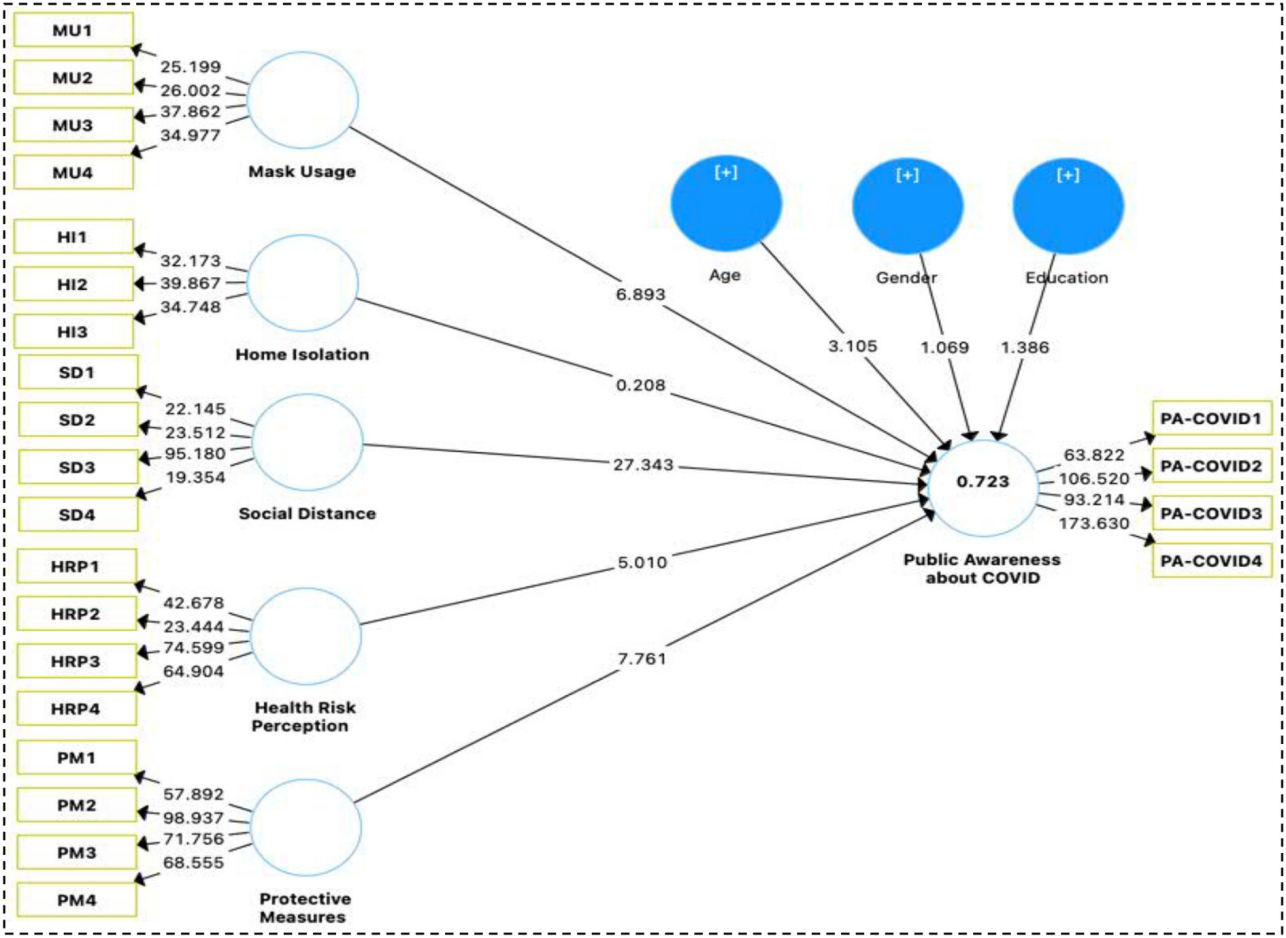
PLS-SEM path model.

Step two of PLS-SEM analysis entails checking out the structural model. Consider the structural path model's predictive relevance, multicollinearity, empirical importance of the route coefficients, and confidence level, among other things. Further, the structural route model's trustworthiness must be assessed. Using the recommendations from Hair et al. (43), this research analyzed the data and made the model's efficacy. We've put a model through its paces to look into how different variables affect PA.COVID in a causal sense. In conclusion, the PLS-SEM path analysis findings (Figure 4) showed an *R*^2^-value of 0.723, an adjusted *R*^2^-value of 0.721, and a *Q*^2^-value of 0.581, indicating that our model is fit (Table 4).

**Table 4:** Path analysis (PLS-SEM).

**Statistical paths**	**Beta (β)**	**Std. dev**	***T*** **-value**	***P*** **-value**	**Hypothesis**
HRP -> PA-COVID	−0.145	0.029	5.101	0.000	Accepted
HI -> PA-COVID	−0.006	0.028	0.208	0.835	Rejected
MU -> PA-COVID	0.185	0.027	6.893	0.000	Accepted
PM -> PA-COVID	0.260	0.034	7.761	0.000	Accepted
SD -> PA-COVID	0.638	0.023	27.343	0.000	Accepted
**Control variables**					
Age -> PA-COVID	−0.054	0.017	3.105	0.002	Significant
Gender -> PA-COVID	−0.020	0.019	1.069	0.285	Insignificant
Edu -> PA-COVID	−0.025	0.018	1.386	0.166	Insignificant
*R* ^2^	0.723				
Adjusted *R*^2^	0.721				
*Q* ^2^	0.581				

**Figure 5:**
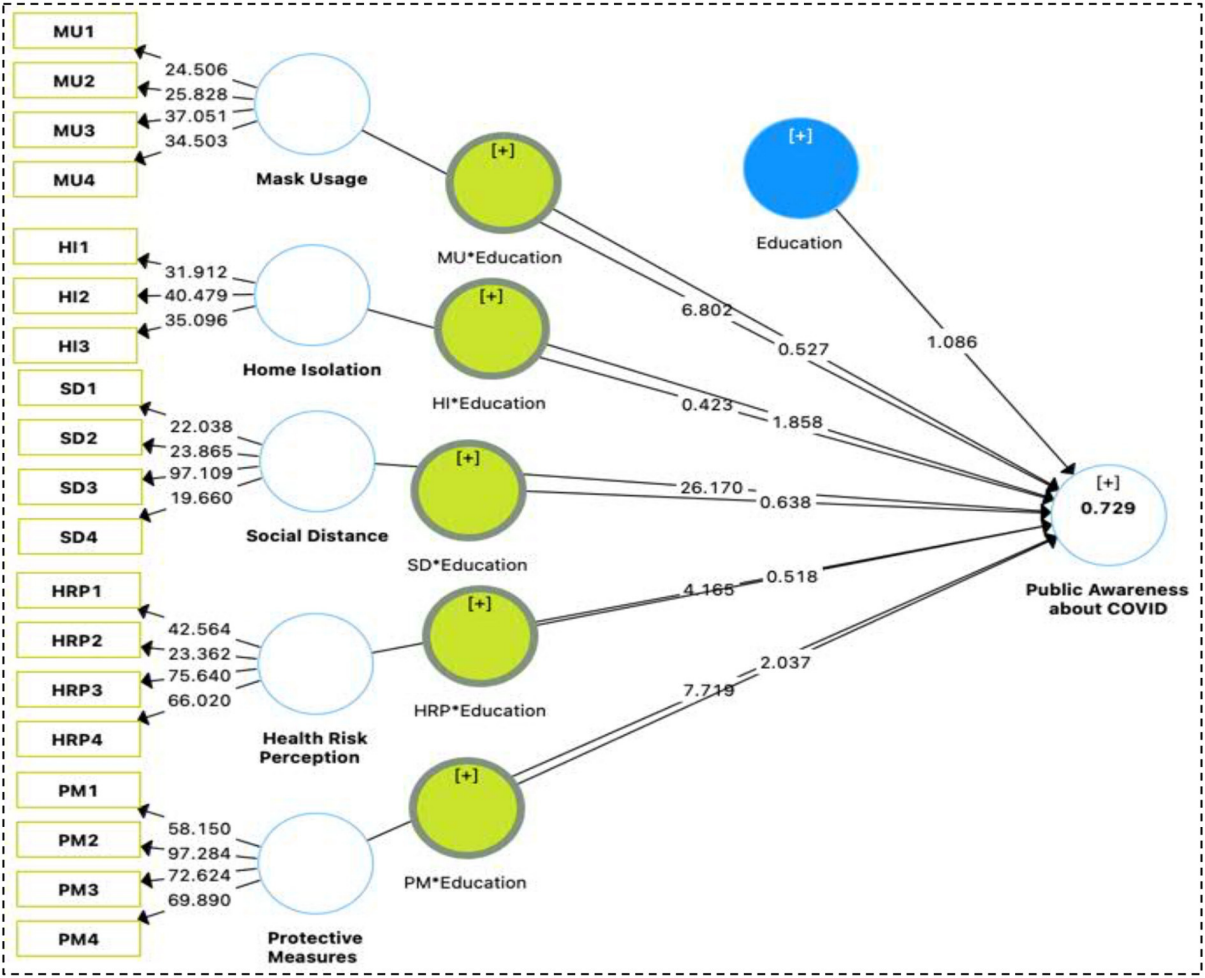
Moderator (education).

We started by looking at the known causal linkages between the different variables to see if they matched up with the hypotheses that had been proposed Mask Use (MU), Personal Hygiene (PH), Social Distance (SD), Risk Perception (RP), Protective Measures (PM), and Public Awareness of COVID-19 were all examined using PLS-SEM to determine any correlations between the variables (PA-COVID). After that, we performed a bootstrapping test with 5,000 repeats to see how well our results matched the hypothesis (40). PLS-SEM direct path analysis revealed HRP -> PA-COVID (β = −0.145, *p* < 0.000) HI -> PA-COVID (β = −0.006, *p* < 0.835), MU -> PA-COVID (β = 0.185, *p* < 0.000), PM -> PA-COVID (β = 0.260, *p* < 0.000), SD -> PA-COVID (β = 0.638, *p* < 0.000). These findings provide credence to the acceptance of hypotheses H1, H3, and H5, but reject H2. We have also considered demographic factors such as respondents' ages, genders, and levels of education. A person's age is a positive control variable, and an older and more experienced individual may be more conscious of COVID and its influence on independent variables, however, neither gender nor level of education had any significant association with PA-COVID (Table 5 and Figure 4). The last step of the PLS-SEM study consisted of determining whether or not there were statistically significantly different between the groups of users' awareness of COVID. Categorical moderation is assessed in PLS-SEM so that we can investigate hypotheses ranging from H1a through H5a. We ran the second model by adding the moderation effect of gender and applied the same settings of bootstrapping with 5,000 sample repetitions. Study results revealed a small moderation effect in the relationships between understudy independent and dependent variables. Education significantly moderates the relationship of PA-COVID associated with Mask Usage (MU), Personal Hygienic (PH), Social Distance (SD), Risk Perception (RP), Protective Measures (PM), Public Awareness about COVID-19 (PA-COVID), depicts the moderation role of education on the relationship between MU^*^Education->PA-COVID (β = 0.013, *p* < 0.598), HI^*^Education->PA.COVID (β = −0.058, *p* < 0.0.063), SD^*^Education->PA.COVID (β = 0.015, *p* < 0.524), HRP^*^Education->PA.COVID (β = 0.016, *p* < 0.605), PM^*^Education -> PA.COVID (β = 0.068, *p* < 0.042). Under this research, H5a has significant relationships with education as a moderator while H1a-H4a did not show any relationship with the moderator (Table 5 and Figure 5).

**Table 5:** Moderation effect.

**Statistical paths**	**Beta (β)**	**Std. dev**	***T*** **-value**	***P*** **-value**	**Hypothesis**
MU*Edu -> PA-COVID	0.013	0.026	0.527	0.598	Rejected
HI*Edu -> PA-COVID	−0.058	0.031	1.858	0.063	Rejected
SD*Edu -> PA-COVID	0.015	0.023	0.638	0.524	Rejected
HRP*Edu -> PA-COVID	0.016	0.030	0.518	0.605	Rejected
PM*Edu -> PA-COVID	0.068	0.034	2.037	0.042	Accepted

Mathematics models can depict a realistic connection between the described parameters. As well as illuminating the structure of the interrelationships between the variables, this analysis allows for the generation of forecasts. The multivariate parameters were analyzed with Pearson's correlation, and the results show a strong relationship between them as shown by the highlighted values. Everything has a positive association, ranging from strong to mild. Age, gender, education, Mask Usage (MU), Personal Hygiene (PH), Social Distance (SD), Risk Perception (RP), Protective Measures (PM), and Public Awareness of COVID-19 are displayed in Table 6 along with descriptive statistics and correlation coefficients (PA-COVID). The correlation test is a method for examining the connection between two or more independent variables. There is no value outside of a +1.00 correlation or a – 1.00 value. The larger the number, the stronger the association, with 1 being the strongest. There is a moderate to a strong relationship between the variables in this study, and the correlation values among the variables that were chosen are statistically significant. All of the variables were statistically significant, and there was a weak to moderate degree of connection between the ones we chose (Table 5).

**Table 6:** Correlation among variable.

**Variables**	**Age**	**Edu**	**Gender**	**HRP**	**HI**	**MU**	**PM**	**PA-COVID**	**SD**
Age	1.000								
Edu	0.164	1.000							
Gender	−0.207	−0.216	1.000						
HRP	−0.032	0.177	−0.125	1.000					
HI	−0.112	−0.038	−0.006	0.316	1.000				
MU	0.041	0.016	−0.056	0.191	0.315	1.000			
PM	−0.054	0.167	−0.068	0.657	0.369	0.319	1.000		
PA-COVID	−0.130	−0.066	−0.000	0.234	0.394	0.544	0.511	1.000	
SD	−0.119	−0.091	0.021	0.272	0.444	0.479	0.450	0.809	1.000

### Artificial neural network (ANN)

**Figure 6:**
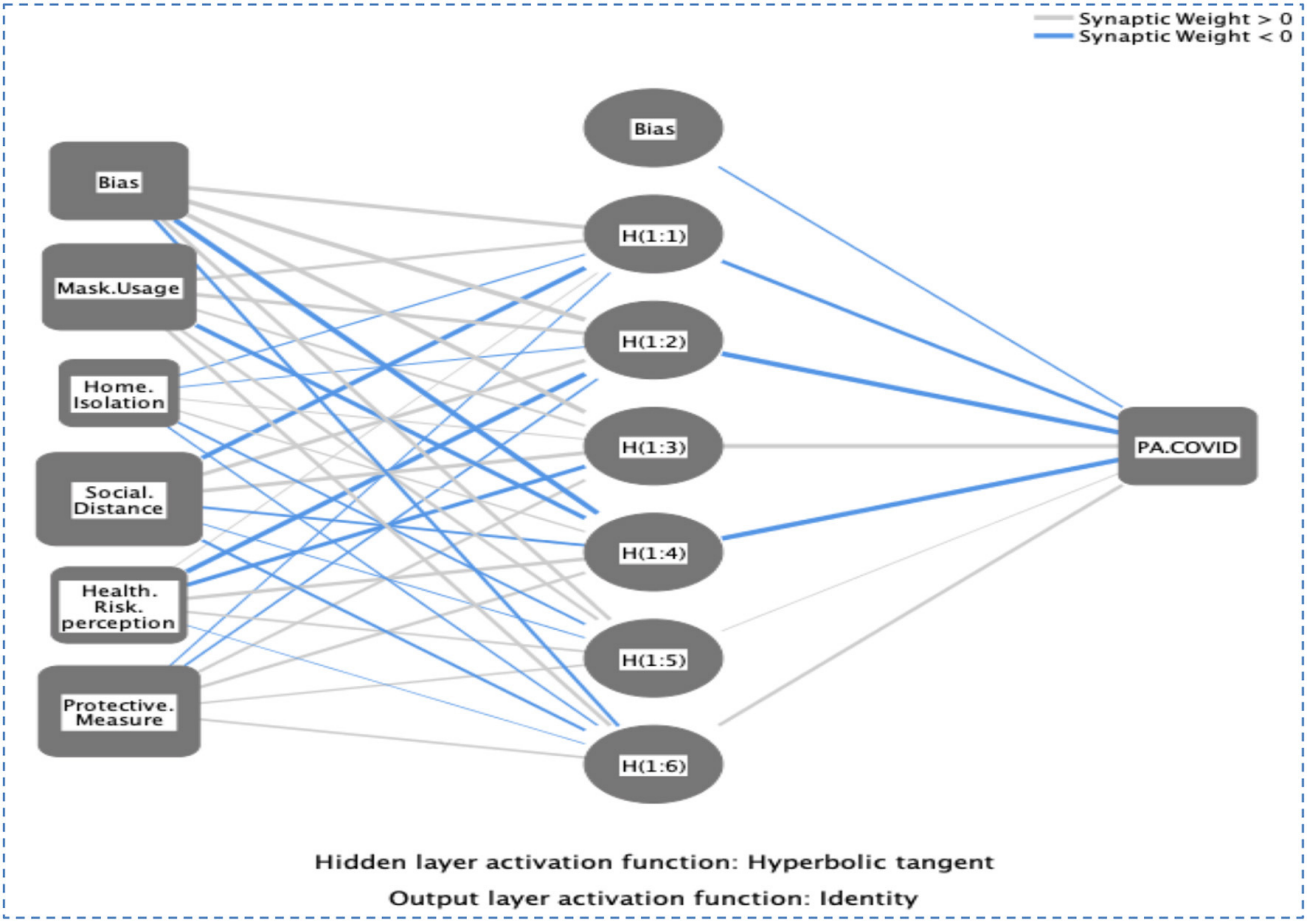
ANN sketch for COVID.

**Figure 7:**
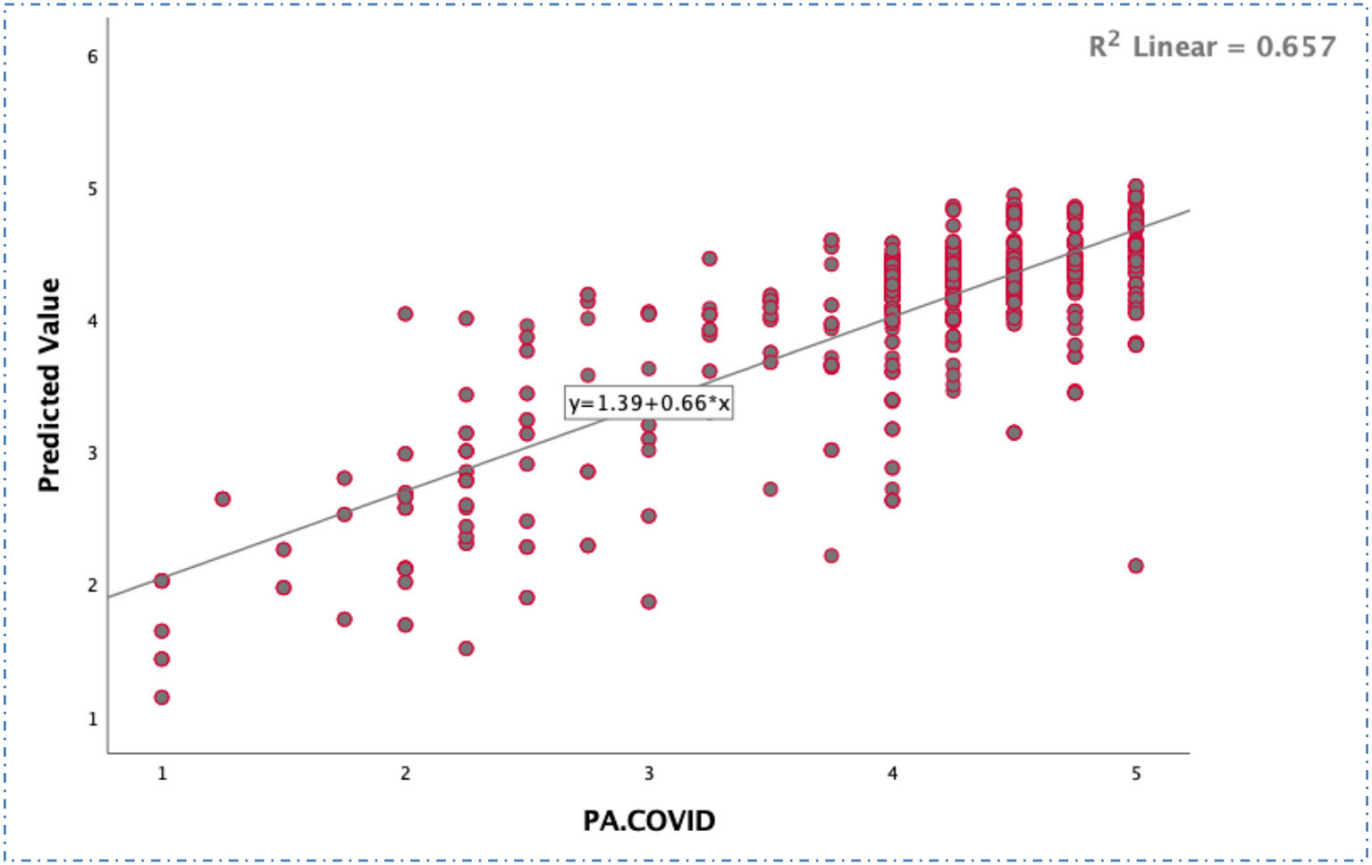
Regression standard residuals for the COVID model.

Software that can simulate non-linear statistical data is called an “artificial neural network,” or “ANN.” Several training sessions might improve its performance (39–41). ANN may be used to not only predict but also organize large amounts of data. When compared to other multivariate models, the ANN model excels in making predictions. Because of this, professionals think it may be used in tandem with SEM to get a more precise outcome. For this reason, ANN is especially helpful when working with little data or flawed assumptions, as it does not rely on multivariate assumptions (39–41). The artificial neural network (ANN) model we've developed for spreading information about COVID-19 (PA-COVID) follows in the footsteps of previous studies (39–41). The sigmoid, whose activation function was selected, hides two layers of neurons from view (Figure 6). Only 30% of the data was used for the actual testing, whereas 70% was used for actual training (Table 7). The root means square of the errors is displayed in Table 7 (RMSE). Validity measures how well a model can predict a certain result. With RMSE values of 0.424 for training and 0.394 for testing, we observed that our ANN model for public awareness of COVID-19 (PA-COVID) had a strong predictive ability. In addition, a specific approach was used to construct a goodness-of-fit coefficient to evaluate the performance of the ANN models. Regression models use a coefficient called *R*^2^ with a value similar to this (Figure 7).

**Table 7:** RMSE values for training and testing.

**Training**			**Testing**			
***N***	**SSE**	**RMSE**	***N***	**SSE**	**RMSE**	**Total sample**
661	118.963	0.424	259	40.189	0.394	920

R^2^ = 1-RMSE/S^2^, where S^2^ is the variance of the test data's desired output and *N*, number of samples; RMSE, root mean square of errors.

### Sensitivity analysis

Table 8 displays the results of a sensitivity analysis performed using an ANN model. The non-zero synaptic weight values that were hidden in the ANN model demonstrated the inputs' significance. This proof relied on the fact that the inputs were non-zero. The model's output is highly sensitive to changes in the input values used to calculate the “relative importance” of each component. The model's output is sensitive to the values assigned to its inputs. After making these observations, we performed a ratio computation to determine the normalized significance of each variable relative to the value found to be the greatest in total. This allowed us to get the most crucial factors in order. Using these contrasts, we were able to evaluate the importance of separate factors about the whole. Based on the sensitivity analysis results, we determined that PA.COVID had the highest relative normalized relevance for our sample (100%, Table 8). These factors were then followed by MU (54.6%), HI (11.1%), SD (100.0%), HRP (28.5%), and PM (64.6%) were likewise shown to be the least important factors for consumers in developing countries struggling with diseases caused by contaminated water. In addition, a specific approach was used to construct a goodness-of-fit coefficient to evaluate the performance of the ANN models. Regression models use a coefficient called *R*^2^ (*R*^2^ Linear = 0.657) with a value similar to this (Figure 7).

**Table 8:** Sensitivity analysis (ANN model for PA-COVID).

**Neural network**	**MU**	**HI**	**SD**	**HRP**	**PM**
NN	54.6%	11.1%	100.0%	28.5%	64.6%

MU, Mask Usage; PH, Personal Hygienic; SD, Social Distance; RP, Risk Perception; PM, Protective Measures; PA-COVID, Public Awareness about COVID-19.

## Conclusion

We have applied SEM-ANN dual-stage hybrid approach to conclude the results. PLS-SEM was applied to check the relationship among variables. To test the validity of the hypotheses that had been put up previously, First, we had a look at the established connections between the various factors. The direct route analysis of PLS-SEM confirms the validity of hypotheses H1, H3, and H5, while also confirming the validity of H2. Additionally, we have accounted for the respondents' age, gender, and level of education as a kind of control. While gender and level of education did not correlate with PA-COVID, age did, suggesting that age may serve as a protective control variable and that a more seasoned individual may have a greater understanding of COVID and its effects on independent variables. Study results revealed a small moderation effect in the relationships between understudy independent and dependent variables. Education significantly moderates the relationship of PA-COVID associate MU, PH, SD, RP, PM, PA-COVID, depicts the moderation role of education on the relationship between MU^*^Education->PA-COVID, HI^*^Education->PA.COVID, SD^*^Education->PA.COVID, HRP^*^Education->PA.COVID, PM^*^Education -> PA.COVID. Under this research, H5a has significant relationships with education as a moderator while H1a-H4a did not show any relationship with a moderator. The artificial neural network (ANN) model we've developed for spreading information about COVID-19 (PA-COVID) follows in the footsteps of previous studies. The root means the square of the errors (RMSE). Validity measures how well a model can predict a certain result. With RMSE values of 0.424 for training and 0.394 for testing, we observed that our ANN model for public awareness of COVID-19 (PA-COVID) had a strong predictive ability. Based on the sensitivity analysis results, we determined that PA. COVID had the highest relative normalized relevance for our sample (100%). These factors were then followed by MU (54.6%), HI (11.1%), SD (100.0%), HRP (28.5%), and PM (64.6%) were likewise shown to be the least important factors for consumers in developing countries struggling with diseases caused by contaminated water. In addition, a specific approach was used to construct a goodness-of-fit coefficient to evaluate the performance of the ANN models. Regression models use a coefficient called *R*^2^ (*R*^2^ Linear = 0.657) with a value similar to this. We have concluded that awareness about COVID is most important against COVID infection. Many statistical tools were used to check the relationship among different variables. There is a moderate to strong relationship between the variables in this study, and the correlation values among the variables that were chosen are statistically significant. Only one district of Punjab Province, Pakistan, was included in the analysis. This study's major findings, however, can be used elsewhere. The research will be useful in advocating for better public health monitoring and policy.

## Data availability statement

The original contributions presented in the study are included in the article/supplementary material, further inquiries can be directed to the corresponding author/s.

## Author contributions

MS: conceptualization, methodology, software, and writing-original draft. PM: supervision, final draft approval, data collection, analyzing, editing, and data collection. MY and SM: visualization and investigation. All authors contributed to the article and approved the submitted version.

## Funding

This study was supported by the Internal Research Project Excellence 2022 at the Faculty of Informatics and Management, University of Hradec Kralove, Czech Republic.

## Conflict of interest

The authors declare that the research was conducted in the absence of any commercial or financial relationships that could be construed as a potential conflict of interest.

## Publisher's note

All claims expressed in this article are solely those of the authors and do not necessarily represent those of their affiliated organizations, or those of the publisher, the editors and the reviewers. Any product that may be evaluated in this article, or claim that may be made by its manufacturer, is not guaranteed or endorsed by the publisher.
